# Fundamental Concepts of Human Thermoregulation and Adaptation to Heat: A Review in the Context of Global Warming

**DOI:** 10.3390/ijerph17217795

**Published:** 2020-10-24

**Authors:** Chin Leong Lim

**Affiliations:** Lee Kong Chian School of Medicine, Nanyang Technological University, 11 Mandalay Road, Singapore 308232, Singapore; fabianlim@ntu.edu.sg

**Keywords:** thermoregulation, global warming, heat, work, exercise, hydration, fluid, acclimatization, acclimation

## Abstract

The international community has recognized global warming as an impending catastrophe that poses significant threat to life on earth. In response, the signatories of the Paris Agreement (2015) have committed to limit the increase in global mean temperature to <1.5 °C from pre-industry period, which is defined as 1850–1890. Considering that the protection of human life is a central focus in the Paris Agreement, the naturally endowed properties of the human body to protect itself from environmental extremes should form the core of an integrated and multifaceted solution against global warming. Scholars believe that heat and thermoregulation played important roles in the evolution of life and continue to be a central mechanism that allows humans to explore, labor and live in extreme conditions. However, the international effort against global warming has focused primarily on protecting the environment and on the reduction of greenhouse gases by changing human behavior, industrial practices and government policies, with limited consideration given to the nature and design of the human thermoregulatory system. Global warming is projected to challenge the limits of human thermoregulation, which can be enhanced by complementing innate human thermo-plasticity with the appropriate behavioral changes and technological innovations. Therefore, the primary aim of this review is to discuss the fundamental concepts and physiology of human thermoregulation as the underlying bases for human adaptation to global warming. Potential strategies to extend human tolerance against environmental heat through behavioral adaptations and technological innovations will also be discussed. An important behavioral adaptation postulated by this review is that sleep/wake cycles would gravitate towards a sub-nocturnal pattern, especially for outdoor activities, to avoid the heat in the day. Technologically, the current concept of air conditioning the space in the room would likely steer towards the concept of targeted body surface cooling. The current review was conducted using materials that were derived from PubMed search engine and the personal library of the author. The PubMed search was conducted using combinations of keywords that are related to the theme and topics in the respective sections of the review. The final set of articles selected were considered “state of the art,” based on their contributions to the strength of scientific evidence and novelty in the domain knowledge on human thermoregulation and global warming.

## 1. Introduction

The ability to sense, respond and adapt to external environmental threats is one of the key attributes that supported the evolution of life, from a single protocell unit to vertebrates, spanning about 500 million years [1]. This innate property of thermo-plasticity, though limited in extent, forms the underlying physiological functions that allow humans to explore, live, socialize, and labor under extremes of environmental temperature (Tev) [2]. Some scholars postulated that heat provided the energy that drove the organization and formation of protocells from polypeptides, which evolved into unicellular and multicellular organisms over time [3,4,5]. Even as life evolved from multicellular organisms into biological organs and systems, the ability to regulate temperature within a physiological range remains a key requirement for survival and daily functions [2]. Although primitive by time of existence, the thermoregulatory system continues to play vital roles in supporting human life and daily functions in present time [6].

Body temperature (Tb) is regulated centrally by the brain to function within a narrow range of about 3–4 °C, from a resting Tb of ≈ 36.8 °C [7,8]. Scholars subscribe to the multi-century consensus that the upper limit for normal Tb is about 40 °C [7,9], but evidence derived from cancer patients [10,11] and endurance runners [12,13,14,15] showed that humans can tolerate a higher core temperature (Tc) of up to 42 °C without any health consequences. Within this Tb range, the thermoregulatory system also exhibits some degree of thermo-plasticity that is capable of semi-permanent adaptation to heat through the process of heat acclimatization (HA) [2,16,17,18]. Daily exposure to moderate intensity work under sub-lethal heat stress for up to 14 days enhances the heat dissipation mechanisms to result in lower Tb under the same workload and environmental condition [9,19,20,21,22,23]. These adaptations can be reversed with prolonged absence of heat and exercise exposures [18,24]. Long term passive exposure to warm and humid weather conditions, such as natives in tropical regions, also induces some degree of HA [20,25]. The highly adaptable attributes of Tb regulation has played critical roles in allowing man to survive and function in the heat since the evolution of mankind [1,2,5], but its limits are likely to be challenged by the impending threat of global warming in the near future.

The international community recognizes global warming as an impending catastrophe that will cause irreversible damage to the environment and also threatens life on earth [26,27]. The unprecedented rate and scale of destruction on the environment is so severe in the last century that scientists acknowledged the beginning of new geological epoch, known as the Anthropocene age, which replaces the current Holocene age that existed for 11,700 years before it was formally adopted in 1885 [28]. The Kyoto Protocol in 1999 was the earliest international forum to formally recognize the catastrophic impact of global warming and to call for actions to limit the increase in global mean temperature (GMT) to <2 °C above pre-industrial period; the pre-industrial period was defined later by the Inter-government Panel on Climate Change (IPCC) to be 1850–1890 [28]. Through the effort of the United Nations Framework Convention on Climate Change, 195 countries became signatories of the Paris Agreement in 2015, which reinforced the urgent need to limit global warming to <2 °C above pre-industrial period [28]. A target of <1.5 °C increase in GMT above pre-industrial period was set by the Paris Agreement as the “defense line” from the limit of 2 °C. An increase in GMT by >2 °C above pre-industrial period was forecasted to have far reaching impact on the environment and to cause irreversible destruction of earth’s natural geography that would lead to food and water scarcity and extreme weather conditions [27,29]. GMT has been increasing since the beginning of the century with industrialization [28]. The first decade of year 2000 was labelled by the World Meteorological Services as the decade of extreme hot conditions [30,31] and 2014 was the hottest year in the history of meteorological monitoring [32]. GMT between the periods 1850–1890 and 2006–2015 increased at a rate of 0.87 °C/decade and 0.91 °C/decade [28,33], respectively. This rate of global warming would breach the 2 °C threshold by 2040. Readers are referred to a special report from IPCC for comprehensive information on this topic [34].

The weather condition is ubiquitous with human existence, behavior, and functions [35]. For example, the type of dressing, design of homes and buildings, sleep–wake cycles, and the profile of daily activities are influence by seasonal changes in weather conditions [36,37,38]. Global warming would have even greater impact on those who engage in physical work in the outdoors, such as laborers, farmers, military personnel, and fire-fighters [39,40,41,42,43,44,45]. Military personnel and fire-fighters are exposed to additional heat load because of the uniforms and protective suits they work in and the physical load they carry [16,21,25,40,42,46,47,48]. Outdoor sports, especially those performed at high intensity over longer duration would also be impeded by global warming [12,13,49,50,51,52,53,54,55,56,57]. Metabolic heat production can increase by >10-fold during high intensity physical exertion, which can overwhelm the heat dissipation mechanisms and promote heat storage in the body [6,7]. Global warming would have wide ranging impact on human health, functions and activities, which in turn, would have downstream effects on the operation and design of economies, industries and societies [27]. Human-centricity is, therefore, an imperative in the design of strategies and solutions to limit the effects of global warming on life and planet earth.

The key strategies for meeting the target set at the Paris Agreement have focused primarily on reducing greenhouse gases through changes in human and social behaviors, industry practices and government policies [29,34,58]. While such effort is important to mitigate the increase in GMT, limited attention has been given to the physiology of the thermoregulatory system, to protect humans from the effects of a higher Tev [59]. Other than measures aimed solely at protecting the environment, any potential solution to protect humans from the effects of a warmer environment would need to interface with the innate thermoregulatory system. Given this background, the primary aim of this review is to discuss the fundamental concepts and physiology of human thermoregulation as the underlying bases for human adaptation to global warming. Part one will discuss the fundamental concepts of human thermoregulation and the physiological mechanisms that drive human adaptation to a warmer environment. Part two will focus on potential strategies for adapting to a warmer environment through innate thermoregulatory mechanisms, and in combination with behavioral adaptations and technological innovations. The current review was conducted using materials that were derived from PubMed search engine and the personal library of the author. The PubMed search was conducted using combinations of keywords that are related to the overall theme and topics in the respective sections of the review. The final set of articles selected were considered “state of the art” based on their contributions to the strength of scientific evidence and novelty in the domain knowledge on human thermoregulation and global warming.

## 2. Part I: Fundamental Concepts and Physiology of Human Thermoregulation

Part I will revisit some key concepts of human thermoregulation and the physiological mechanisms that drive both acute and chronic adaptations to heat. A basic understanding of this domain knowledge provides the background and a consistent framework for appreciating the discussion on adaptation to global warming in Part II.

### 2.1. Definitions and Indicators of Thermoregulation

This section will define key terminologies that are commonly used in thermoregulation research. Tb is the general term used to describe the state of heat storage in the body and mean Tb was defined as the composite of core (Tc, 64%) and skin (Tsk, 37%) temperatures [1,7]. Mean Tsk is estimated from the composite of body-surface temperatures measured at the chest (30%), arm (30%), thigh (20%) and leg (20%) [60]. However, these indicators of Tb are seldom used in thermoregulation research mainly because Tsk is subjected to the influenced of Tev and not Tb alone [7,61,62,63,64]. Tc, on the other hand, is regulated centrally by the brain to respond in a controlled manner to changes in thermal balance (heat production, absorption and dissipation) within the body [1,8]. For more than a century, Tc has been used as the main Tb indicator for diagnosing fever, defining hypothermia and hyperthermia, and for indicating the associated risk of heat and cold injuries [7,63].

#### 2.1.1. Core Temperature

Tc refers to the deep body temperature in the internal environment of the body, i.e., the abdominal, thoracic, and cranial cavities [1,8]. From a measurement perspective, Tc refers to the temperature of venous blood returning to the heart, which stores excess metabolic heat produced in the organs [65,66,67]. The temperature of venous blood and afferent signals from thermo-sensitive nerves on the body surface are used by the brain as reference temperatures for regulating autonomic and behavioral responses around a Tb set-point [8]. As the measurement of venous blood temperature is invasive and impractical to perform in research and clinical settings, the most common sites for indirect measurement of Tc are the rectum, esophagus, and gastrointestinal tract [68,69,70,71] for research, and the axillar, sub-lingual, ear canal, and forehead for diagnosis of fever [7]. In this review, and unless specified, Tc is used generically to encompass temperatures measured in the sites used for research [1,7,72,73].

#### 2.1.2. Heat Tolerance

Heat tolerance was defined as cellular adaptation caused by a single sublethal heat exposure that allows the organism to survive a subsequent exposure to lethal heat stress [74]. This definition of heat tolerance was based on the protective effects of heat shock proteins on cellular structures against lethal heat stress, following a single dose of exposure to sub-lethal heat stress a.k.a. heat shock response. However, the heat shock response was never subjected to human experimentation due to the need for exposure to lethal heat stress. This review takes a “whole-human” approach and defines heat tolerance as the ability to tolerate heat stress without physiological and work failures. Physiological or work failures could range from the inability to sustain workload (i.e., fatigue) to the occurrence of heat illness. Such a definition of heat tolerance is applicable to public health, occupational, and sport settings.

#### 2.1.3. Heat Strain and Heat Stress

Heat strain and heat stress are used interchangeably sometimes to describe a state of positive heat storage in the body, although these are different indexes of thermoregulation. From a physiological perspective, a “strain” refers to a stretch or departure from an original state of existence. Therefore, heat strain is defined as the magnitude of departure from resting Tc, i.e., difference between peak and resting Tc. The physiological concept of “stress,” on the other hand, refers to the sum of physiological demands for an adaptation to maintain homeostasis and to protect the survival of the host [75]. Consistent with this concept, heat stress is defined as the total heat load that the thermoregulatory system has to cope with to maintain physiological homeostasis and is indicated by the peak Tc. For example, if Tc increased from 37 °C to 39.8 °C during a 10-km run, heat strain would be 2.8 °C and heat stress would be 39.8 °C. Therefore, an improvement in heat tolerance would be due mainly to the ability to tolerate a higher level of heat stress and not necessarily due to a higher level of heat strain tolerance.

#### 2.1.4. Rate of Rise in Core Temperature

Although there is no formal consensus among scholars, the efficacy of the thermoregulatory system can be indicated by the acuteness of the Tc response curve during exercise and heat exposure, i.e., rate of rise in Tc (RORtc). RORtc is the sum of the balance between the rate of heat production, absorption and dissipation. Heat accumulation is due to metabolic heat production and heat absorbed from the environment [6,7,8]. Heat dissipation, on the other hand, is driven by the transfer of heat from the skin to the environment through the processes of evaporation, conduction, convection and radiation [6,7,8], which will be discussed in the next section. RORtc is calculated by dividing heat strain (change in Tc) by the time of exposure to the stimulus (e.g., heat and physical work) that caused Tc to increase. Tc would increase at a faster rate if the rate of heat accumulation is faster than the rate of heat dissipation, and vice-versa. Therefore, a useful indication of positive heat adaptation or more effective thermoregulatory function is a slower RORtc during heat and physical work exposures. The main effect of a slower RORtc is an increase in work duration before reaching the Tc limit for heat tolerance, without changing the limits of the Tc threshold for heat tolerance.

### 2.2. The Physical Properties of Heat Transfer and Storage

The net amount of heat stored in the body is a function of the balance between heat gain and loss. This thermal balance is driven the amount of metabolic heat produced, heat exchange between the skin and the environment through conduction convection, and radiation, as well as through evaporative heat loss. Heat transfer between the skin and the environment is bi-directional and down the temperature gradient for conductive, convective and radiation heat exchanges. Heat transfer is unidirectional for evaporative heat loss, moving from the skin to the environment, and the magnitude of evaporative heat loss is inversely associated with the water vapor pressure in the environment. Metabolic heat production, on the other hand, only adds heat to the body because energy is produced continuously to sustain life. The sum of these channels of heat transfer and production determines the state of thermal balance in the body, which can be expressed in the following equation [6,8]:

Heat Storage = + M ± Ra ± Cv ± Cd − E (1)

where M = metabolic heat production, Ra = radiative heat exchange, Cv = convective heat exchange, Cd = conductive heat exchange, and E = heat loss due to evaporation.

#### 2.2.1. Metabolic Heat Production

The energy produced in the metabolic process is stored in adenosine triphosphate (ATP). When energy is needed to sustain basal metabolic rate or to perform physical work, one of the phosphate bonds in ATP is split to result in a unit of phosphate and an adenosine diphosphate (ADP). The splitting of the phosphate bond produces about 7.3 Kcal of heat, which is harnessed as energy to drive muscle contraction to produce mechanical work [76]. However, only about 25–30% of metabolic heat produced is used by muscles to perform mechanical work [77,78]. The remaining >70% of metabolic heat is stored in the body with no physiological functions, which explains the increase in body temperature when performing physical work [79,80]. The excess metabolic heat needs to be removed from the muscles to maintain intramuscular thermal balance and to prevent thermolysis of muscle cells. As heat is transferred down the temperature gradient, the excess metabolic heat in the muscle is transferred to the cooler arterial blood flowing into the capillary bed [80] and stored in the venous blood flowing out of the muscle. Heat stored in venous blood is circulated back to the heart and conserved within the body to promote heat storage or transported to the skin surface for dissipation into the environment [81]. Because the metabolic system functions continuously to meet energy demands in the body and heat is a byproduct of metabolism, metabolic heat production is always positive in the heat storage equation, i.e., only adds heat to the body. During intense exercise, metabolic rate can increase acutely by >10-fold [6,80], which is an important attribute for meeting energy demands to increase work output quickly. However, an acute increase in metabolic heat production also puts a strain on the thermoregulatory system to maintain thermal balance [78,82,83]. During prolonged intense physical work, metabolic heat production can outweigh heat removal and Tc can increase to >40 °C, even in cool conditions [15,84,85,86]. The moderation of metabolic heat production and an increase in the rate of heat removal can have significant impact on thermoregulation homeostasis during physical work in hot environment.

#### 2.2.2. Radiative Heat Exchange

Radiative heat exchange refers to the physical transfer of heat between two non-contacting surfaces through the movement of heat in the air. In humans, radiative heat exchange occurs between the skin and the environment and the direction of heat transfer depends on the gradient between Tsk and Tev [6,7]. In the outdoors, the main source of radiative heat is the sun [87], which can be trapped in, as well as reflected from, ground and building surfaces to people in the environment [6,38,88,89]. Heat-producing machineries, such as compressors for air-conditioner, car engines, and power generators, can also be sources of radiative heat transfer from the environment to people in the surrounding. Performing physical work under direct sunlight or near to heat-producing machineries would promote radiative heat transfer from the environment to the skin. On the other hand, the skin can lose heat to the environment through radiation if the Tsk is higher than Tev, such as during exposure to winter conditions or when sitting in a cold room. Exposure to radiative heat from the environment is expected to increase significantly under global warming conditions. Besides behavioral changes, the design of the urban environment can also help to moderate radiative heat exposure from the environment [35,38]. More than half the world population currently live in cities that are classified as urban heat islands (UHI) [90], and global warming is expected to increase the intensity and impact of UHI [91]. UHI refers to the higher Tev in cities than rural areas due to urbanization, and a significant source of UHI is heat radiated from building and road surfaces [92,93]. An increase in vegetation and trees in the environment and changes to the materials used for pavements and buildings can potentially help to reduce the UHI effect by shading the heat emitted from these surfaces [90]. However, the effectiveness of these measures in coping with the full impact of global warming remains unknown currently.

#### 2.2.3. Conductive Heat Exchange

Conductive heat exchange occurs when heat is transferred through contact between two static surfaces [8]. This form of heat exchange is also bi-directional and down the heat gradient, from the warmer to the cooler surface. In occupational settings, conductive heat exchange can occur between the surfaces of heat-emitting equipment and the skin [7], such as communication and electronic equipment carried by soldiers and outdoor workers. In contrast, Conductive heat exchange provides an important channel for rapid removal of body heat when resuscitating heat injury victims using cold-water immersion, because of the acute gradient between Tsk (>37.5 °C) and cold water temperature (~4 °C) [94,95]. During winter, the use of electric thermal blankets conducts heat from the surface of the blanket to the skin to maintain Tb at a comfort zone. The same concept of the thermal blanket may be used for reverse application in the context of global warming, by installing cooling mechanisms in sofas, beds, blankets, and mattresses to keep the body cool through conductive heat transfer (discussed later). This approach to body cooling would be more efficient than the current air conditioning (AC) systems, which emit heat and carbon to environment and may not be sustainable under global warming conditions [96,97,98].

#### 2.2.4. Convective Heat Exchange

Convective heat exchange refers to the transfer of heat by a heat-trapping medium (water or air) flowing across a static surface. This avenue of heat exchange is also bi-directional and involves the transfer of heat between the skin and water or air moving across the surface of the body [7]. When taking a shower, for example, the skin absorbs heat from the water if Tsk is lower than water temperature, and vice-versa. The same principle applies when an electric fan directs the flow of wind against the surface of the body, which implies that the fan is only useful for body cooling when Tsk <Tev. Blowing warm air against cooler skin results in the transfer of heat from the air to the skin through convection. For this reason, the fan is not an effective mode of cooling during heat waves, when Tev is usually higher than Tsk [99,100,101], and might be redundant as a stand-alone body cooling tool under the global warming conditions. Despite the Paris Agreement, the GMT is projected to increase by 3–5 °C by the year 2100 [102], which would potentially reverse convective heat exchange, driving the transfer of heat from the environment to the skin. These anticipated extreme conditions should drive future innovations to design clothing with convective cooling capabilities and to develop body cooling systems to sustain outdoor work and recreation [96,103]. Clearly, there is tremendous need for transformation in body cooling technologies to keep pace with the rate of global warming.

#### 2.2.5. Evaporative Heat Loss

Evaporative heat loss occurs when the physical state of liquid undergoes expansion and changes to gaseous state [6]. The expansion of the liquid allows heat trapped in moisture to be dissipated to the environment. This form of heat loss is unidirectional, where heat stored in the liquid can only be transferred to the environment and not the other way around [7]. In humans, evaporative heat loss occurs primarily through the evaporation of sweat, which accounts for up to 80% of heat dissipation during intense exercise, making it the primary channel of heat dissipation [6,104,105]. The extent and rate of evaporative heat loss is inversely associated with the water vapor pressure (moisture content) gradient between the skin surface and the air [106]. In thermoregulation research, relative humidity (Rh) is commonly used as a surrogate indicator of moisture content in the air.

The physiological function of sweat production is critical for promoting evaporative heat loss to preserve thermal balance. During exercise and heat exposures, some portion of venous blood that stores excess metabolic heat needs to be transported to the skin to induce sweating. The rate of sweat loss can range from about 1 L/h to 3.5 L/h, depending on body weight, sweat gland volume and activity, work intensity, climate, and state of HA [104,105,107,108,109,110]. Although sweating is the primary innate body cooling mechanism, sweating alone, without evaporation, results in the loss of body fluid, with minimal heat loss [104]. Therefore, the evaporation process is essential for significant heat loss to be derived from sweating. However, because the fluid in sweat is derived from plasma in circulating blood [107,108,110], prolonged excessive sweating without adequate fluid replacement can result in the depletion of blood volume [45,82,111,112,113]. Blood volume depletion leads to a reduction in cardiac output and blood flow to the skin and muscle, which in turn compromise heat dissipation and work output [111,113,114,115]. At the extreme, blood circulation can be interrupted due to a mismatch between venous return, cardiac output and the demand for blood flow, which can lead to fainting [112,116,117,118,119]. Therefore, the cost of sweating and evaporative heat loss is borne by the cardiovascular system (CVS) and the fluid balance mechanisms, due to the need to defend blood volume against sweat loss. The topic of fluid replacement during exercise in the heat will be discussed in more detail later.

Since evaporative heat loss is inversely related to the volume of moisture in the air, the extent of evaporative loss can be promoted by having a high sweat rate and low Rh [17,120]. Conversely, a high Rh or low sweat rate can independently impede evaporative heat loss and promote heat storage, independent of Tev [121,122]. This dissociation between heat storage and Tev explains the potential occurrence of hyperthermia (a.k.a. incompensable heat stress) and heat-related injuries when undertaking manual tasks in cool but humid conditions [14,104,123,124,125]. The same principle implies that a high Rh can limit the effectiveness of clothing materials that claim to have superior wicking properties to promote heat dissipation. The wicking property of the material can only promote the transfer of sweat from skin to the external environment, but the extent of evaporative heat loss would still depend on the amount of moisture content on the air [61,126]. Global warming would likely expose inhabitants in the tropical region to the dual-threat of high Tev and Rh, because Rh is inherently high in these regions. For example, the Rh in Singapore ranges from about 60% in the noon to >95% at about 0200 h in the morning daily throughout the year [12,13,51,127,128]. These developments are likely to challenge the limits of evaporative heat loss mechanisms, which would need to be reinforced with behavioral and technological solutions to preserve thermoregulation homeostasis under the effects of global warming.

### 2.3. The Physiology of Thermoregulation and Adaptation

The physical attributes of heat transfer inform us on the channels of heat transfer between the body and the external environment. The next section will explain the mechanisms regulating the body’s responses and adaptations to heat in both the acute and chronic timeframes.

#### 2.3.1. Central Regulation of Body Temperature

**Figure 1:**
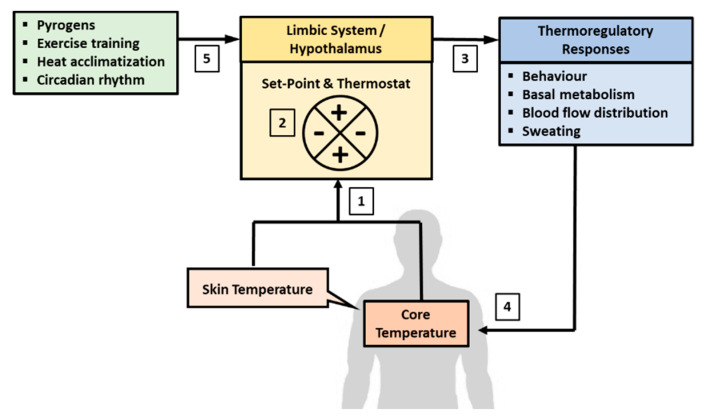
Central regulation of body temperature. Body temperature is regulated autonomously in the limbic system, which includes the hypothalamus. (1) The brain receives afferent signals on the state of body temperature from core (Tc) and skin (Tsk) temperatures. Tc is sensed from temperature of blood flowing to the brain and Tsk is derived from thermal sensitive nerves distribute all over the surface of the body. (2) The signals from Tsk and Tc are matched against the temperature set-point, which is about 36.8 °C in resting condition. (3) A departure from the set-point would activate a “thermostat” response through the autonomic nervous system to recalibrate body temperature back to the set-point. (4) The recalibration of body temperature may involve changes in behavior, blood flow distribution, basal metabolic rate adjustments, and the induction of sweating if heat loss is needed. (5) The temperature set-point can also fluctuate due to the influence of circadian rhythm, adaption to physical training, heat acclimatization, and pyrogens.

Humans are both endotherms and homeotherms by nature. The endothermic property refers to the ability to produce heat endogenously (heat gain) through the metabolic pathway and the homeothermic property refers to the innate regulation of heat gain and loss to maintain homeostasis of Tb. The endothermic property acts in concert with the physical channels of heat transfer (heat absorption or loss), and as part of the homeothermic processes, to achieve homeostasis of Tb [1]. Both the endothermic and homeothermic functions are coordinated centrally through a Tc set-point by a “thermostat” mechanism in the hypothalamus, which is part of the limbic system in the brain [1,8,129,130] (Figure 1). The limbic system regulates emotions and motivation, which drives behavior to avoid pain and to seek reward. The hypothalamus is part of the limbic system and serves as common point of consolidation for efferent outputs to preserve physiological homeostasis through autonomic regulation [129,131,132]. The central thermostat receives afferent feedback on Tev from thermal-sensitive nerves distributed all over the surface of the body (i.e., Tsk), and feedback on Tc from blood flowing to the brain [133]. This feedback mechanism demonstrates the dual-thermic property of human thermoregulation, which comprises the shell (Tsk) and the core (Tc) [1]. Signals from Tsk and Tc are integrated and matched against a Tc set-point that is regulated centrally in the brain [8].

Under a resting state, Tc is regulated at around 36.8 ± 0.5 °C and this set-point can be adjusted with HA and physical training, and by endogenous pyrogens [8,9,134,135]. The Tc set-point also fluctuates with circadian rhythm, decreasing to about 36.5–36.8 °C during sleep and increasing to about 37–37.5 °C in the wakeful hours of the day [136,137]. Any departure from the tolerance limit of the Tc set-point would trigger a “thermostat” response to recalibrate Tc through alterations in behavior and physiological responses induced by the autonomic nervous system (ANS) [8]. For example, when Tc falls below the set-point, the ANS would increase metabolic heat production (endothermic property) and induce peripheral vasoconstriction to conserve heat in the core of the body (homeothermic property) [138]. The cold sensation also promotes heat-conservation behavior, such as to increase clothing insulation and to seek warmer environment [2,138,139]. In contrast, when Tc increases above the set-point, the ANS would stimulate peripheral vasodilation and channel more blood to the skin surface to dissipate heat to the environment [132,133,140]. Higher Tb also promotes heat dissipating behavior to seek cooler environment and to reduce insulation over the skin [141,142]. Physiologically, the skin plays important roles in Tb homeostasis by conserving heat (vasoconstriction) when Tc is below the set-point and by promoting heat dissipation (vasodilation) when Tc is higher than the set-point. In this regard, shell temperature is said to be slave to core temperature [1].

#### 2.3.2. Central Regulation of Body Temperature during Physical Work

The performance of physical work in sport and occupational settings can increases metabolic heat production by >10-fold, which imposes additional stress on the central regulation of Tb. In response, the ANS would channel more blood to the skin to induce sweating and to increase heat dissipation to the environment [23,81]. During intense exercise, the thermoregulatory mechanisms can also act centrally to decrease metabolic heat production by moderating work rate to keep Tc within a range that is physiologically tolerable [143]. For example, running performance in a half-marathon in tropical climate (WBGT 26–29.2 °C) was inversely related to Tc response [12], with the slower runners having higher peak Tc during the race. In the same half-marathon race conducted three years later, running pace, and not the hydration status, was found to be the main determinant of Tc response [13]. A laboratory experiment found no difference in an 8-km time trial performance between African (27.4 ± 1 min) and Caucasian (27.4 ± 0.4 min) runners in cool condition (15 °C, 15% relative humidity, Rh), but the African runners completed the time-trial >3 min faster than the Caucasian runners under hot condition (35 °C, 60% Rh) [144]. The better running performance among the African runners in the heat was attributed to the slower rate of heat storage, due to their smaller physique and lighter body mass, compared with the Caucasian runners. These results demonstrated the ergogenic properties of Tb regulation for endurance performance and the importance of maintaining thermal balance during physical performance in the heat [145,146,147].

The current evidence suggests that the association between Tb regulation and physical performance is regulated centrally to protect the body from the ramifications of “overheating” [148]. The Critical Tc Theory proposed that the central mechanisms of thermoregulation protect the body from “overheating” by impeding work performance at a critical Tc threshold [143,146,149,150,151]. For example, a group of trained cyclists performed separate trials that manipulated their starting Tc and RORtc, but volitional fatigue occurred consistently in all trials when Tc was 40.01–40.03 °C [151]. The achievement of this Tc threshold corresponded with the decrease in stroke volume and increase in heart rate (HR), suggesting that the central fatigue mechanism was induced by compromising cardiac functions [151]. Some scholars also subscribe to the concept of the Central Governor Theory, which proposed that peripheral feedback during exercise allows the brain to anticipate a catastrophic event and to act in advance to protect the body from danger [143]. For example, in the study mentioned above, there was no difference in 8-km time trial performance between African and Caucasian runners in cool condition, but the Caucasian runners performed the same run 3 min slower than the African runners in hot condition [144]. The difference in running performance was attributed to higher heat storage in the Caucasian runners due to their higher body masses, which caused the central governor mechanism to slow down their running paces (and heat production) to avoid the ramifications of “overheating” [143]. The inverse relationship between running pace and Tc in half-marathons also supports the concept of the Central Governor Theory [12,13]. The evidence presented reiterates the central regulation of Tb, not only to preserve homeostasis of Tb, but also to protect homeostasis of the body as a whole organism against the threshold of heat tolerance. A consequence of this regulatory mechanism is the modulation of physical work output to match the rate of heat production with the rate of heat dissipation, i.e., preservation of thermal balance within the limits of heat tolerance. Therefore, improvements in thermoregulation can be an effective strategy for improving work output (heat tolerance) in both cool and hot conditions [85,152,153].

In summary, Tb is regulated autonomously throughout the day, around the set-point of 36.8 ± 0.5 °C, by maintaining the balance between heat dissipating and conserving mechanisms and through alterations in Tb-related behavior. Examples of Tb-related behavior include choice of clothes, places of social and occupational activities, daily routines and time of activities, and the use of AC and heating systems to achieve thermal comfort [140,154,155]. During intense exercise, Tc can increase by 3–4 °C from resting level, and the sound regulation of RORtc is achieved by balancing the rates of heat dissipation and metabolic heat production. Work is impeded centrally when Tc reaches a “critical” level, possibly as a protective mechanism against the effects of hyperthermia. This evidence demonstrates the critical role of the thermoregulatory system, as an innate mechanism that is centrally regulated to support humans’ survival and functions in the environment that we operate in [2]. 

#### 2.3.3. Cardiovascular Stress, Fluid Homeostasis and Thermoregulation

**Figure 2:**
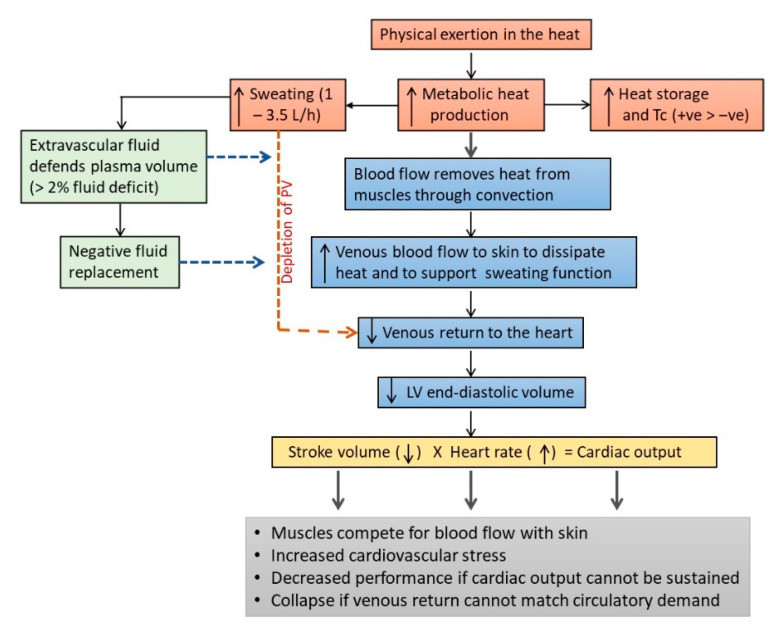
Effects of physical work and exercise on fluid homeostasis and cardiovascular (CVS) functions. Intense physical work can increase metabolic heat production by >10-fold, leading to an increase in heat storage and the activation of the sweating response to dissipate heat. Metabolic heat produced in the muscle is transferred to venous blood and transported to the skin to be dissipated to the environment. The diversion of venous blood to the skin reduces the volume of venous blood returning to the heart, leading to lower stroke volume and a higher stress load on CVS to maintain cardiac output and to meet the demand for muscle blood flow. The increased demand on the CVS is further burdened by the loss of plasma volume (PV) due to sweating, which can range about 1–3.5 L/h. The loss of PV can be defended by the influx of fluid from extravascular compartments, for up to about 2% loss of body weight. Beyond this level, and in the absence of adequate fluid replacement, excessive sweating can deplete PV, leading to lower blood volume to meet the demands of skin and muscle blood flow. Under these circumstances, physical work would need to slow down or cease due to the decrease in cardiac output. At the extreme, fainting may occur due to insufficient blood flow to the brain. LV = left ventricle and Tc = core temperature.

The two physiological systems that “pay the price” for maintaining Tb during intense exercise in the heat are the CVS and the fluid balance mechanisms (Figure 2). The higher demand on these systems results mainly from the diversion of venous blood to the skin for heat dissipation and the loss of plasma volume through sweating [81,156]. As mentioned earlier, a portion of venous blood is diverted to the skin for heat dissipation during intense exercise, which reduces the volume of venous blood returning to the heart, and which lead eventually to a decrease in stroke volume [113,115]. Although the reduction in stroke volume can be defended to some extent by increasing HR, this mechanism to maintain cardiac output is insufficient to meet the demand for muscle blood flow during prolonged physical work in the heat [112,116,157,158,159,160]. Therefore, the muscle and skin compete for blood flow during physical work in the heat and the burden of this competition is borne mainly by the CVS, which has to maintain cardiac output with less venous blood returning to the heart [112,116,161]. The higher demand that exercising in the heat imposes on the CVS was shown in the HR and Tc responses when running at 70% of maximum volume of oxygen uptake (VO_2max_) for 60 min in cool (25 °C, 60% Rh) and warm (35 °C, 50% Rh) conditions [85]. Mean Tc at the end of the run was 38.4 °C in cool condition and 39.1 °C in warm condition, which corresponded with an 11.4% higher mean HR in the warm (176 bpm), compared with the cool (158 bpm) condition. Tc did not differ between the two conditions in the first 20 min of the run, but HR in the warmer condition began increasing faster than the cool condition after about 5 min of running, indicating cardiovascular compensation to meet blood flow demands for heat dissipation and physical work output in the warm condition.

The effects of a lower venous return are compounded by the loss of plasma volume due to sweating, which can range from about 1 L/h to 3.5 L/h [25,116]. Excessive sweating in the absence of adequate fluid replacement can lead to a decrease in blood volume, which compounds the stress on the compensatory mechanisms in the CVS to preserve cardiac output [113]. For example, compared with a state of euhydration, a 1% dehydration during moderate intensity exercise (60% VO_2peak_) decreased stroke volume by 9 mL, with a corresponding 10 bpm increase in HR [117]. The stress on the CVS increased further with 3% dehydration, which decreased stroke volume by 18 mL and increased HR by 18 bpm [117]. Humans are largely aqueous creatures, with 60% of body mass attributed to water stored in intracellular, interstitial (intercellular) and intravascular (blood circulation) compartments [143]. In a state of dehydration, fluid from the extravascular compartments can be shifted into the intravascular space to preserve blood volume, but this compensatory response can only protect blood volume for an equivalent of about 2–3% of body weight loss, before the effects of dehydration and exhaustion set in [112,115,116,162]. It was estimated that 1 L of fluid deficit during physical work in the heat would result in 1 L/min decrease in cardiac output [163] and a 4% dehydration would decrease cardiac output by 13%, with a corresponding 5% decrease in mean arterial pressure [112]. A shrinking blood volume also leads to a reduction in sweat rate, which further compromises heat dissipation [116,151]. When exacerbated, the gap between arterial and venous blood flow can widen to the point that is physiologically intolerable, where exercise is forced to cease or fainting may occur due to insufficient blood supply to the brain [143,158].

Fainting at the extreme of dehydration is a self-limiting mechanism that prevents further depletion of blood volume by forcing the cessation of physical work and by lowering metabolic heat production. This self-limiting mechanism under a state of dehydration may contribute to the occurrence of heat exhaustion (HE) and may also explain the absence of fatality due to dehydration in sport, occupational and social settings. The only dehydration-related fatality that this author is aware of involved three cases of intentional rapid weight loss among collegiate wrestlers. The wrestlers lost about 10% of body weight (10–12 kg) through similar dehydration regimes over 10–14 weeks in an attempt to compete in lower bodyweight categories and they all died of cardiac arrest [164]. These cases involved extreme level of self-induced dehydration that were prolonged over >10 weeks, which are not representative of the nature and extent of dehydration that occurs in sport and occupational settings. In the absence of other health conditions, such as heat stroke (HS) or cardiac arrest, fainting due to dehydration is usually reversible by lifting the legs above the heart level (~12 inches), in supine position, to restore blood circulation [143,158,165]. Although maintaining a state of euhydration contributes to better thermoregulation during exercise, this function is secondary to the primary aim of defending blood volume and maintaining muscle and skin blood flow. Evidence from a half-marathon race that resulted in 2–6% of fluid deficit showed that Tb was associated with running pace and not with fluid intake or sweat loss [13]. Body weight loss of 1–8% that occurred during marathons also had no negative effects on Tb [166]. These results concurred with an earlier study showing that running faster than training pace during races, and not the state of hydration, was the main cause of heat injury in 20 novice road race participants [167]. This evidence supports the notion that Tc and heat tolerance during exercise is driven primarily by work intensity, and that some degree of dehydration is ptolerable during prolonged intense physical work. Current consensus on fluid intake accepts that mild dehydration of 2–3% of body weight loss is well-tolerated by most individuals during performance of physical work and that fluid intake should be driven by thirst and the need to avoid >2% loss in body weight loss [143,162,168]. The volume of fluid deficit that is not replaced during work performance can be replaced gradually during the recovery period. Global warming would increase the demand for fluid replacement when performing manual tasks in the outdoors, due to higher sweat rate. In public health, occupational and sport settings, it is important to empower the stakeholders with greater awareness on the need, purpose and appropriate approach to hydration. Such empowerment would enable individuals to take responsibility to remain well-hydrated when undertaking physically demanding tasks in the heat.

#### 2.3.4. Sex-Related Differences in Thermoregulation

From the perspective of anthropometry and under a constant workload, a higher body surface area (BSA) and body mass (BM) and a lower BSA/BM ratio are beneficial for maintaining thermal balance [169]. Compared with men, women generally have lower BSA and body mass, and a higher BSA/BM ratio [170] and fat content for each kg of body mass [20]. Women also have lower sweat rate than men for the same amount of metabolic heat production [171,172]. These anthropometric and sweating characteristics predispose women to a higher degree of heat storage than men when undertaking physical work at the same absolute workload [173]. In a controlled experiment, where active men and women performed the same absolute workload on a cycle ergometer for 60 min in hot-wet (35 °C, 80% Rh) and hot-dry (45 °C, 20% Rh) conditions, in separate trials, the female subjects recorded significantly higher rectal temperature (Tre, 0.2–0.3 °C) and %VO_2max_ (21–39%) than male subjects in both conditions [170]. These differences in Tre persisted even after correcting for VO_2max_ and could be contributed partly by the higher relative workload (%VO_2max_) in the female subjects. Therefore, when absolute workload is held constant, women are likely to have a higher level of heat stress than men due to differences in anthropometry and a higher relative workload intensity. Furthermore, the risk of having symptoms that are associated with orthostatic intolerance is five-fold higher in women than in men during intense exercise in the heat [174]. Orthostatic intolerance is due primarily to a mismatch between venous and demand for cardiac output.

However, anthropometric factors may influence thermoregulation differently when work intensity is adjusted to individual levels. For example, during a 40-km race in hot and humid environment, highly trained women runners had lower Tre than male runners in the last 10 km (0.7 °C) and during recovery (1.1 °C) [175]. The lower Tre in the female runners is likely due to the lower metabolic rate that was associated with a lower body mass (69.5 kg in males runners and 53.9 kg in female runners) and a slower running pace of about 10 min over the 40 km race. There was no difference in fluid intake, hydration status, relative running intensity and heart rate between male and female runners during the race. Studies conducted on male runners also reported that running pace was the main determinant of Tc [13] and that a lower rate of heat production due to lower body mass was beneficial for endurance performance [144]. Therefore, the combination of a lower body mass and work intensity can reduce the magnitude of heat production and storage in both male and female athletes. Compared with male athletes, the lower body mass in female athletes would have a lower propensity for heat storage, especially when combined with a lower work intensity. Women also have lower Tc than men when exercising under hot-wet condition because of the higher BSA/BM ratio and lower sweat rate [176]. In contrast, having a higher sweat rate resulted in lower Tc in men, compared with women, when exercising in hot-dry condition [170,176].

The higher propensity for heat storage in women due to anthropometric characteristics did not translate to higher risk of heat illness than men. A meta-analysis of 22 studies found that the overall risk for heat illness was 2.64 times higher in men than in women, and the risk of heat-related mortality was 1.89 higher in men compared with women [177]. These data reiterated the multifaceted risk of heat illness that is not influenced by heat storage alone. For example, the higher likelihood of men to be involved in physical work in hot environments (e.g., sport and military and fore service personnel) than women were not accounted for in these studies. However, there is a difference in the rate of adaptation to HA between male and female subjects. In young active males, resting (−0.24 °C) and peak (−0.36 °C) Tre and peak heart rate (−14 bpm) improved significantly in the first five days of HA, with no further improvements observed when HA was extended for another five days [178]. This profile of adaptation was reversed in female subjects, who showed no significant changes in the same parameters measured in the first five days of HA, but had significantly lower resting (−0.22 °C) and peak (−0.41 °C) Tre, and peak heart rate (−10 bpm) when HA was extended for another five days [178]. The reasons for the different profiles of adaptation to HA between male and female subjects are unclear, but could be associated with lower basal and exercise metabolic rates in the female subjects, which would have moderated the level of heat stress exposure for the same period of HA, leading to a slower rate of adaptation.

The data presented suggest that sex-related differences in anthropometry may predispose women to a higher level of heat stress than men when undertaking the same absolute workload. However, when work intensity is individualized, having a lower body mass may be beneficial for maintaining thermal balance in women, especially when combined with a lower intensity of work. Current evidence also suggests that women may require a longer period of HA to induce the same magnitude of thermoregulatory adaptations as man.

#### 2.3.5. Food Intake Behavior in Hot Environment

There is general agreement that performing physical work in hot environment would shift the mix of metabolic fuel to lower fat and higher carbohydrate (CHO) utilization [179]. These findings imply that besides fluid intake, there may be a need to increase CHO intake during prolonged period of exposure to physical exertion in the heat. However, both exercise and heat exposures lead to suppression of hormonal signals for promoting food intake and total energy intake. For example, an acute bout of exercise and the combination of overfeeding and exercise, without heat exposure, suppressed food intake by decreasing the circulating concentration of ghrelin and by increasing the concentrations of pancreatic peptide (PP), cholecystokinin (CKK), peptide tyrosine tyrosine (peptide YY), and glucogon-like peptide-1 (GLP-1) in the blood [180,181,182]. These biomarkers are diet regulating hormones that promote (ghrelin) or suppress (PP, CKK, peptide YY and GLP-1) the signals for food intake. Another study showed that exercising in the heat increased the concentration of circulating peptide YY, with no effect observed on dietary intake [183].

However, results from laboratory studies did not show any association between energy intake, exercise, and Tev. For example, male subjects who rested and cycled at 60% of VO_2max_ in cool (22 °C) and warm (31 °C) environments for 40 min, in four separate trials, had the same amount of absolute energy intake during the ad libitum meal provided 30 min after each experimental condition [184]. In that study, exercising in the heat suppressed plasma ghrelin concentration, but had no effect on diet-suppressing hormones and total energy intake. The 40-min of exposure to the different experimental conditions may be too short to induce significant changes in energy intake and responses in diet-suppressing hormones. In another controlled experiment, military personnel were subjected to 2 h of physical exercise in hot (30 °C), thermoneutral (21 °C), and cold (−10 °C) conditions and to 8 h of rest in thermoneutral condition (21 °C), in four separate trials, where they were allowed ad libitum consumption of the standard combat ration [185]. In all the four conditions, the study participants consumed 70% of the combat ration, resulting in similar amount of energy intake (1920–1985 Kcal). The equivocal energy intake across the experimental conditions could be influenced by consumption habits of combat ration that were developed prior to the study.

The impact of Tev and physical exertion on energy intake was more obvious in field conditions. Special Operation Forces personnel operating in warm-humid (Tev 27 ± 2 °C, Rh 66.8 ± 8.7%) and cold-humid (Tev 9.3 ± 3.5 °C, Rh 71.5 ± 13.6%) conditions, on separate missions, ingested only 52% of average total energy expenditure (4618 Kcal) [186]. Although there was no significant difference in daily energy expenditure between the two conditions, average energy intake in hot-humid condition (2200 Kcal) was lower by about 27%, compared with cold-humid condition (3001 Kcal). These results showed that physical exertion alone can suppress energy intake, but the magnitude of energy intake suppression was greater in hot-humid than in cold-humid conditions, even in well-trained military personnel.

In summary, there appears to be a mismatch between energy demand and intake during physical exertion alone or in combination with hot environment. Laboratory studies showed no association between diet-regulating hormones, environmental conditions, and energy intake, with exercise alone or in combination with cold to warm environmental conditions. However, evidence from a field study showed significant energy deficit in both hot and cold conditions, with a greater extent of energy intake suppression in hot condition. This evidence also implies the need to further investigate the potential health consequences of prolonged caloric deficit in sport and occupational settings that involve prolonged exposure to physical exertion, especially in hot conditions.

The discussion in Part I highlighted the importance of thermoregulation for human survival and daily functions. Homeostasis of thermoregulation is achieved through physical channels of heat transfer between the skin and the environment, and through the central regulation of the body temperature in the brain around a thermostatic set-point. The primary role of physical heat transfer is to maintain thermal balance through the removal of endogenous heat from the body to the environment (in a state of positive thermal balance), or through endogenous heat production and the absorption of exogenous heat from the environment into the body (in a state of negative thermal balance). The primary drivers of physical heat transfer are temperature and water vapor gradients between the skin and the environment. The state of thermal balance resulting from physical heat transfer (Tc, state of heat storage) triggers the response of the physiological mechanisms to remove or conserve heat to protect the central temperature set-point. In a state of positive thermal balance (hyperthermia), the CVS would respond by channeling more blood to the skin to promote heat dissipation and to increase cardiac output to meet demand for peripheral blood supply. Central fatigue mechanisms may also be activated to reduce endogenous heat production. In a state of negative heat balance (hypothermia), the physiological mechanisms would inhibit cutaneous blood flow and promote endogenous heat production. In both states of hyperthermia and hypothermia, the physical and physiological mechanisms of thermoregulation are driven towards a common destination of achieving a state of thermal balance, which would coincide with the centrally regulated temperature set-point. The CVS and the fluid balance mechanisms play critical roles in supporting heat removal and conservation by meeting the demands for blood flow and distribution, sweat production and protection of blood volume. Therefore, the homeostasis of thermoregulation is driven in concert by the physical properties of heat transfer between the skin and the environment and by the central regulation of temperature set-point.

## 3. Part II: Potential Strategies for Adaptation to Global Warming

The fundamental concepts and physiology of human thermoregulation discussed in Part I provided the foundation knowledge for Part II, which will briefly discuss the impact of global warming on human life and will focus on potential strategies to cope with a higher level of environmental heat. There is consensus that the capacity of the innate thermoregulation system to adapt and cope with a higher level of Tev would be challenged under global warming conditions and that further adaptation would need to be complemented with behavioral adaptations and technological innovations [20,96]. Due to the futuristic nature of this discussion, some of the adaptation strategies suggested in this review would inevitably be postulations in predicting human adaptation to global warming.

### 3.1. Impact of Global Warming on Human Life and Functions

Despite the commitments made among the signatories of the Paris Agreement, the current projection is that GMT is on course to increase by 3 °C to 5 °C by the year 2100 [102]. If left uncontrolled, global warming would have devasting and irreversible impact on the environment and human life [187]. The impact on the environment include soil degradation, loss of biodiversity, destruction of fresh water resources and ecosystems, acidification of the ocean, rising sea level, higher frequency of hurricanes, floods, and droughts, and the reduction of land that can be used for agriculture [28,34,58]. It was forecasted that 64% of world population would live in water-stressed areas by 2025 [188], and an increase in GMT by 2.5 °C would put 20–30% of plants and animals at risk of extinction [187]. About 74% of land on earth would also be exposed to a substantial increase in the duration and frequency of wildlife fire seasons [27]. The downstream effects of natural disasters and challenges related to the environment would also increase the exposure of humans to a higher risks of both air- and vector-borne infectious diseases [187,189].

However, the biggest threat that global warming brings to human life would be an exponential increase in heat-related morbidity and mortality, due to higher frequency, intensity and wider area of coverage of heat waves, especially after the year 2100 [58,190]. The current projection is that 420 million of the world population would be exposed to heat waves and another 65 million people to extreme heat waves, if the 2 °C global warming threshold is breached [28,34,58]. It is also worth noting that 20–40% of world population are already living under warmer climates that are >1.5 °C above the pre-industrial period for at least one season annually [34,58]. By 2050, the annual mortality of HS is projected to increase globally by 2.5-fold from the current baseline of 2000 deaths annually [191,192]. Meeting the target set by the Paris Agreement alone does not provide total protection from the health hazard of heat stress. Climate modelling data estimated that the number of countries exposed to the ramifications of heat stress would increase from a baseline of 109, to 129 (1.5 °C warming) and 135 (2 °C warming) countries, or by >15% of land on earth [27] by the year 2100. The data also projected that heat stress would become a significant health hazard in >95% of countries in the world by the end of this century [27]. Climatic modelling using data from 27 cities in China projected an increase in the annual heat-related mortality by 1.5–2-fold (48.8–67.1/million) at 1.5 °C warming of mean surface temperature, and by 1.8–2.5-fold (59.2–81.3/million) at 2 °C warming of mean surface temperature [26]. The combined heat-related mortality among the 831 million inhabitants across the 27 cities would be >28,000 annually if mean surface temperature is increased by 1.5–2 °C [26]. In south Korea, heat related mortality in 2090 was projected to increase by 5.1-fold under a 3–4 °C increase in surface temperature, and by 12.9-fold if surface temperature is increased by >4 °C [29]. In America, Europe, and East Asia, the extreme global warming scenario of >4 °C increase in surface temperature was projected to increase heat-related mortality by 3.5–8.9-fold [193].

These data reiterate that the target set by the Paris Agreement is more likely to moderate, and not to remove, the threats of global warming on human life. Exceeding the Paris Agreement by 1–2 °C in GMT would lead to a much higher magnitude of heat-related mortality across the world. Besides heat-related mortality and morbidity, global warming would also have far-reaching impact in disrupting human functions, especially in outdoor occupational, recreational, and social settings [194]. These potentially devasting projections of global warming strongly support the need to develop strategies to enhance the innate thermoregulatory system, and to exploit the use of behavioral adaptations and technological innovations to further “strengthen” human thermoregulation beyond the naturally endowed mechanisms [20,35,96].

### 3.2. Heat Acclimatization

HA refers to the process of conditioning the body to function in the heat [195] and which demonstrates the property of thermal plasticity in human thermoregulation [23,153]. HA is also the most established strategy for inducing thermoregulatory adaptations [120,134,196,197] and there is good consensus that HA can improve sport performance and work tolerance in the heat [9,135,153,197]. Heat acclimation refers to the similar process conducted in an environmental chamber and HA refers to heat conditioning conducted in the outdoor environment [2]. This review will use the term “heat acclimatization” to refer both forms of heat conditioning.

HA involves daily exposure to submaximal work in the heat for up to 14 days [18,24,153,197] and the physiological adaptations to HA can be observed after 4–6 days, before plateauing off after 10–14 days [23,24,196,197,198,199]. Physiological adaptation to HA, to varying extent, include lower Tc at rest and during exercise, earlier onset of sweating, and increased plasma volume, cardiac output, sweat rate, and VO_2max_ [1,16,18,23,196,200]. For example, a 9-day HA program conducted on 17 military personnel resulted in earlier onset of sweating, higher sweat rate, and volume (40%), lower Tc at rest (−0.4 °C) and after four work-cycles (−0.3 °C), and lower resting (−19 bpm, or −19%) and exercise (−13 bpm or −8%) HR [201]. Besides physiological adaptations, HA is also effective in improving work performance in the heat. One study showed that 10 days of HA improved time trial performance by 8% and power output by 5% when cycling under 30 °C Tev and 30% Rh condition [200]. Six consecutive days of hot water immersion (HWI) at 40 °C following training bouts in temperate climate also induced HA adaptations in both trained and recreational athletes [202] and these adaptations were retained for >2 weeks in the trained individuals [203]. In these studies, Tc at the end of exercise decreased by 0.36 °C in endurance-trained subjects and by 0.47 °C in recreationally active subjects [202,203]. Tc at onset of sweating also decreased by 0.22 °C in endurance-trained subjects and by 0.23 °C in recreationally active subjects [202,203]. The form and extent of adaptation to HA are influenced by work intensity and duration, and by the level of heat exposure [21,152,204,205,206]. For example, the average timing for cyclists completing a 44.3 km time-trial in the heat (37 °C) improved from 77 min at baseline to 69 min after 6 days of HA, and to 66 min after 14 days of HA. Although Tc at the end of the time-trials was about 40.2 °C in all three occasions, the power output was higher than baseline (256 W) by 10% at day-6, and by 15% on day-14, which indicated an increase in heat dissipating capacity [207]. The physiological adaptations that are induced by HA are semi-permanent and can be reversed after 2–4 weeks of absence from heat exposure, i.e., decay in HA [18,24,197]. A systematic review and meta-analysis involving 12 studies reported that HR decreased by 2.6%, and Tc decreased by 2.6% for each day of decay in HA [18]. However, the adaptation in sweat rate was not influenced by the decay in HA but more by the duration and intensity of the HA program.

Physical training in cool conditions may improve work tolerance in the heat to some extent, with some or no impact on thermoregulatory functions [9,21,134]. In one study, performing 4 h of step-up exercises under 21 °C for 12 days improved work tolerance in the heat (Tev 34 °C), but had no significant effect on Tc response [135]. In military personnel, eight weeks of aerobic training in cool condition resulted in partial heat adaptation when performing heat stress test in combat uniform, but these adaptations were not observed when the heat stress test was performed in impermeable protective suits [21]. Conversely, newly conscripted military recruits (18–20 years old), who lived in tropical country, were unable to tolerate physical work in the heat, even when Tb was still within physiological range [25]. This evidence indicates that physical training alone can improve performance in the heat, possibly due to improvements in aerobic fitness [208]. However, the combination of physical exertion and heat exposure are essential for inducing the thermoregulatory adaptations. Long term passive exposure to hot conditions may result in some degree of HA, but does not contribute to physical performance in the heat [20,25]. The evidence presented demonstrates the property of thermo-plasticity in human thermoregulation and the consistency of HA as an intervention strategy for inducing physiological adaptations to improve work and heat tolerance. However, the benefits of HA resulting from a higher sweat rate to promote evaporative heat loss would be limited in environments with high Rh e.g., microclimates of protective suits that are used in occupational settings. Other strategies to protect body temperature regulation are needed under these conditions.

#### 3.2.1. Effectiveness of Heat Acclimatization When Working in Impermeable Clothing

The benefits of HA may be limited in sports and occupations that require the donning of clothing that is semi- or fully impermeable to sweat and heat transfer, e.g., fencing and fire protection suits, body armor vests, and chemical defense suits [194,209]. Such clothing forms a microenvironment that impede the physical transfer of heat and the evaporation of sweat between the skin and the environment. For example, 6 and 12 days of HA resulted in lower Tc and HR at rest and during a heat stress test (Tev 40 °C and 30% Rh) in combat attire, and the Tc adaptation was about 1-fold greater in the 12-day, compared with the 6-day HA program [16]. Although both HA programs increased work tolerance time by 11–15%, the Tc and HR adaptations were significantly moderated when the heat stress test was performed in impermeable suits. In a study conducted by this author, 33 military personnel participated in a 14-day HA program and underwent heat stress tests in the environmental chamber (36 °C Tev, 65% Rh and 800 W/m^2^ simulated solar radiation) before, in the middle and after HA [103]. Each heat stress test involved marching at 4 km/h for 3 × 45 min work-cycles with 15 min rest between each cycle. One group of participants performed the heat stress tests in military uniform, carrying the standard battle load (SBL, ~18 kg), and wore the impermeable bullet-proof vest that covered the upper body. Another group performed the heat stress tests wearing the same outfit plus the standard backpack (i.e., full-battle load, FBL, ~35 kg total load). In both groups, the HA program had no effect on Tc response (RORtc 0.028 °C/min in SBL and 0.037 °C in FBL) because heat transfer in the upper body was impeded by the bullet-proof vest. However, resting Tc decreased by 0.2–0.3 °C after HA in both groups, which contributed to higher work tolerance in the heat by 20% in SBL and by 17% in FBL. Furthermore, with the use of a prototype personal cooling device (PCD), work tolerance in the same conditions improved by 2-fold in the SBL group and by 1.2-fold in the FBL group, with Tc remaining <39.5 °C in all three work-cycles. The prototype PCD delivered dry air and circulated chilled fluid into the suit to promote convective and evaporative heat loss. These results reiterated the benefits of HA as a baseline strategy for inducing thermal adaptation and for improving work tolerance in the heat, under conditions where the avenues of dissipation are not impeded. However, in conditions where heat dissipation is significantly impeded, other interventions, such as the PCD, are needed for sustaining physical work in the heat.

#### 3.2.2. Heat Acclimatization and Prevention of Heat Injury

The two most common forms of exertional heat injury (EHI) are HE and HS, which are discussed in greater detail elsewhere [123,210,211,212,213,214]. HE victims usually collapse due to physical exhaustion with Tc of about 40 °C or lower [123,215]. This condition is usually not fatal and most victims would recover with the restoration of blood circulation [123,165,213,214]. HS on the other hand is a fatal form of heat injury that impairs the central nervous system [123,210,216]. Victims of HS have Tc >40 °C and they usually suffer from multi-organ failure and sepsis, which can lead to death and coma [210,211]. There is consensus among scholars that heat is the primary cause of these heat injuries because the victims of HE and HS are consistently hyperthermic [123]. However, these two forms of heat illnesses are not linked mechanistically and clinically, and HS is not an extension of HE. The current evidence also suggests that the role of heat in the mechanisms of HE and HS may not be as significant as previously suggested.

HA is often cited as an important strategy to mitigate the risks of EHI and the lack of HA is cited as a contributing factor to EHI cases in military personnel and athletes [86,123,124,217,218,219]. Although there is consensus that HA improves thermoregulatory functions and work tolerance in the heat, this author is not aware of any direct evidence showing that HA is effective in preventing EHI, especially in HS. Some researchers attributed EHI in military recruits to the lack of HA [39,220], but none of these studies have compared EHI cases between recruits and trained soldiers [217,219] in terms of their HA status. The suggestion that HA is protective against EHI is based on the assumption that heat injury is an extension of hyperthermia [123,217,221]. However, healthy humans could tolerate a Tc of up to 42 °C during physical exertion with no health consequences [12,15,51]. This evidence suggests that EHI occurring <42 °C may be due to other factors and not due to heat alone [12,13,14,210,222].

Although classified as a form of heat illness [123], the mechanism for HE is more likely to be due to a compromise in blood circulation during physical exertion, which can be exacerbated, but not triggered, by heat stress [82,158,213]. The level of Tc reported in HE victims (~40 °C) is physiologically tolerable by healthy individuals [12,13,15], but this level of heat stress may contribute to HE indirectly by imposing greater demands on the CVS. Exercising in the heat causes the diversion of venous blood to skin for sweating, which lowers venous return to the heart. Combined with the potential loss of blood volume due to inadequate fluid replacement, the CVS is challenged to maintain blood flow to the muscle and skin, with a lower cardiac output [113,116]. Most HE victims who lost consciousness are successfully resuscitated by elevating the legs about one foot above the ground to restore blood circulation, [165,213]. These mechanisms of HE suggest that improvements in thermoregulation and aerobic fitness and the increase in plasma volume due to HA can help to preserve blood circulation during intense physical work in the heat, leading to higher tolerance against HE [21,24,153,195]. Therefore, the benefits of HA in lowering the risk of HE is in improving cardiovascular functions to tolerate a higher level of workload in the heat and not by removing the cause of HE. The lower Tc due to HA would also reduce the stress on the CVS, but higher Tc itself does not appear to be the direct cause of HE.

New evidence proposed by the dual pathway model (DPM) suggests that HS is not triggered by heat alone, but is triggered by two independent pathways [210,211]. At Tc <42 °C, HS can be triggered by the heat sepsis, due to exercise- and heat-induced endotoxemia, and the thermolytic effects of heat alone can trigger HS when Tc >42 °C [210,211,212,213,214,215,216,217,218,219,220,221,222,223,224,225,226]. Both pathways of HS can lead to multi-organ failure, central nervous system dysfunction, disseminated intravascular coagulation, and hemorrhage, which are commonly reported in HS victims. The DPM, proposed by this author, argued that exercise and heat stresses increase the permeability of the gut barrier, resulting in the translocation of gram-negative bacteria from the gut into the blood circulation [51,227,228]. These bacteria carry an endotoxin unit (aka lipopolysaccharides, LPS) that is harmful to cells in the body. LPS translocated from the gut is transported by the portal circulation to the liver to be removed from the body. However, LPS can spill into the central circulation when the translocation of LPS overwhelms its removal by the liver [52,229]. In a healthy state, the immune system can remove LPS from the central circulation. However, when the immune system is suppressed, LPS in blood circuation can accumulate to a threshold that could trigger a sepsis response, leading to the clinical presentation observed in HS victims, i.e., endotoxemia pathway of HS [210,211]. Therefore, in the endotoxemia pathway of HS, the role of heat is in inducing the increase in gut barrier permeability, which facilitates LPS translocation, and not in triggering HS. An improvement in thermoregulation may lower the risk of heat sepsis by moderating magnitude of increase in gut barrier permeability, which would impede LPS translocation.

When Tc is >42 °C, the thermolytic effects of heat alone can disintegrate and liquify cellular structures and cause organs to fail, i.e., the second pathway of HS [76,230]. Since the second pathway of HS is triggered by heat, HA can slow down the RORtc to prolong the time taken to achieve the Tc threshold for HS to be triggered. Evidence supporting the DPM includes the protection of animals against lethal heat exposure when LPS was inhibited from entering the circulation, when animals in the control group succumbed of HS [231,232,233]. Healthy runners [12,13,222] and cancer patients exposed to hyperthermia therapy [10] have also tolerated Tc of up to 42 °C with no consequences, and mild endotoxemia has also been observed in healthy runners after endurance races [51,229]. The beneficial effects of HA in lowering the RORtc may help to moderate the increase in gut barrier permeability and impede LPS translocation, and also delay the time taken for Tc to reach the point where the thermolytic effects of heat alone can trigger HS.

In summary, HA can induce beneficial effects on thermoregulation when working under hot conditions. However, the extent of adaptation in the innate thermoregulatory system through HA is limited to a decrease of about 0.3–0.5 °C in Tc during physical work in the heat [18,153,207]. This magnitude of innate adaptation may not be able to cope fully with the projected increase in GMT of 1.5 °C to 5 °C by the end of this century [58,102], especially when performing physical work in the outdoors [20]. The evidence on the effectiveness of HA in improving thermoregulation is also based mostly on single exposure to a heat stress test after a period of HA and is not based on prolonged repeated exposures to heat [18]. The benefits of HA are also impeded when working in microclimates of protective suits, which are impermeable to the transfer of heat and sweat between the skin and the environment. However, because HA induces positive adaptations of the innate thermoregulatory mechanisms, the current evidence supports the use of HA as a baseline strategy to cope with the effects of global warming. Other strategies involving behavioral adaptation and technological innovation can further enhance the functions of the thermoregulatory system.

### 3.3. Circadian Shift to Sub-Nocturnal Lifestyle

Humans are highly adaptable creatures who are driven by instinct to seek comfort and safety to sustain life and daily functions [1,2,141]. In the context of global warming, the fundamental drivers for behavioral adaptation would be to avoid heat and to seek shelter in cooler conditions. These behavioral drivers would translate to remaining indoor during the hot hours of the day and to “hunt” or “socialize” in the outdoors during the cooler hours of the night. Although there are multiple modes of adaptation to achieve such behavior, this review would like to highlight the likelihood of a shift in circadian cycle as a potential strategy, because of its impact on normality of human lifestyle globally. Whether by design or nature, the heat-avoidance behavior would likely lead to shift in circadian cycle towards a sub-nocturnal lifestyle [140,234], especially for populations who are involved in outdoor work and sports, and who do not have access to AC [136,235]. To avoid the heat from the sun, outdoor activities would likely begin closer to, or soon after sunset, and could extend to the next morning before dawn, or soon after sunrise. This projected shift towards a sub-nocturnal lifestyle could be associated with the limbic system, which drives human behavior to operate in a “comfort zone” [141,142,154]. A higher Tev is also associated with sub-optimal mood, mental functions, and motor skill and physical performances, which compromise occupational health and productivity, and sports performance [85,194,236,237,238,239]. An example of circadian-related adaptation to heat in current time is in the planning of the 2020 Tokyo Olympic Games, which was anticipated to have warmer than usual summer climate (The event was postponed to 2021 due to the COVID-19 pandemic). Besides relocating the venue for race walking and marathon from Tokyo to Sapporo (migratory behavior), which is about 5–6 °C cooler, the timing of endurance events (e.g., 5000 m race, marathon, and race walking) was adjusted to the pre-dawn or nights hours to avoid the heat in the day, i.e., circadian shift [240].

The long existence of night shift work in many industries shows that humans can adapt to functioning in the night and to recovering in the day hours. However, the extent of adaptability to a sub-nocturnal lifestyle may vary between individuals, as navy servicemen and healthcare workers showed much inter-individual differences in circadian adaptation during night shifts [241,242,243]. Those who work permanently in night shift showed better adaptation to a nocturnal routine [136,244]. The forces of natural selection would likely dictate that people who are more adaptable to circadian rhythm shift would be more inclined to engage in outdoor work and sports in the night hours [244]. Those who are less capable of adjusting their circadian rhythm would be more likely to engage in indoor sport and labor. Therefore, one potential impact of global warming may be the segregation of human population by lifestyle routine that is driven by the plasticity of the thermoregulatory system and circadian rhythm adaptations.

### 3.4. Mechanization to Reduce Metabolic Heat Production

Given that metabolic heat contributes positively to heat storage, an important area of heat stress management is to limit or remove the need to perform physically demanding tasks, especially in the outdoors. In the contex of global warming, attention should be given to redesigning occupational tasks, by exploring technologies to reduce the need for high metabolic cost. There is already much progress made in promoting the mechanization of manual tasks to enhance productivity and in reducing the reliance on human labor [245,246]. Examples of these human–machine transitions include the use of tools, vehicles, and machines to perform manual work and to transport load across terrains in farming, outdoor labor, and military settings [247,248,249]. Futuristic technologies in robotics and alternative energy are likely to push the current boundaries for mechanization, which would significantly minimize human exposure to high metabolic heat production when undertaking manual tasks [250]. For example, there has been much progress in the development of exoskeletons and robots to assist humans in load carriage [248,249,250,251,252,253], which are applicable in military, agriculture, mining and construction industries. In a laboratory experiment that involved walking with a 23 kg load at 1.5 m/s on a treadmill, the use of battery-powered exoskeleton reduced metabolic cost by an average of 8% [252]. Another study that involved treadmill walking at 1.5 m/s with a 6.8 kg load, the use of a multi-joint soft exoskeleton lowered metabolic costs by 15% to 22%. The technology for exoskeleton is in its infancy, and there is ongoing effort to push the barrier of this technology, especially in the military setting [248]. Given time and technological advances in alternative energy, material science, and artificial intelligence, the technology for exoskeleton would likely advance further towards a wider scale of application in the occupational settings. Technological advances are also being made to enhance human-robot interaction [254] and to develop multi-robot systems to replace human teams in the future [255]. The evidence presented imply that global warming is likely to cause major disruption to occupations that required performance of physical tasks, especially in the outdoors. Besides adjustments in work hours and cycles, the performance of manual tasks would likely shift towards replacement or assistance by machines to protect humans from environmental heat. The role of humans in future occupations would likely focus on generating, and managing intelligent judgements and in dispensing appropriate emotions that cannot be performed by machines.

### 3.5. Solar Energy to Reduce Carbon Emission and Radiative Heat Exposure

Global warming would directly increase human exposure to radiative heat in the environment, due mainly to the effects of greenhouse gases. Carbon emitted from human activities make up more than 95% of greenhouse gases and the surge in carbon emission globally since the industrial age is the main culprit for increasing GMT [34]. One technology that has great potential in reducing radiative heat load in the environment is the trapping and converting radiative heat for use as solar energy [256]. A simulated deployment of solar panels in Paris metropolitan area produced 265 MJ/year/m^2^ of electrical power, which would provide about 28% of domestic energy consumption for heating and AC. Using solar energy also reduced the energy demand for AC by 12% during the summer and lowered Tev in the UHI by 0.2 °C to 0.3 °C [91]. These results demonstrated the potential benefits of solar energy when adopted on a global scale, in reducing carbon and heat emission from AC systems and in reducing the UHI effect. Although solar energy provides less than 1% of total energy consumption currently, the pace of solar energy adoption has accelerated exponentially in the last decade and some scholars projected that solar energy can potentially provide 100% of total energy consumption by 2032 [256]. The optimism in the global adoption of solar energy stems from the lower cost of production, higher efficiency of energy production, and tax incentives given by government agencies to convert from fossil fuel to solar energy [257]. When adopted on a global scale, solar energy technology have great potential in lowering environmental heat and carbon and heat emission from AC systems [91,256]. These benefits of solar energy could lower human exposure to radiative heat, especially when working in the outdoors.

### 3.6. Air and Body Cooling

Global warming is projected to drive the demand for AC exponentially for indoor environment and would provide the impetus for the development of body cooling technologies for both indoor and outdoor environments [98,258]. However, the use of hydrofluorocarbon as refrigerants and the high amount of electrical energy used in AC systems contribute to carbon emission in the environment [97,258]. The compressors in AC systems also produce heat, which contribute directly to the UHI effect [259]. The current global energy consumption from AC is estimated to be about 1 trillion kWh annually [260] and is expected to increase exponentially to 4000 TWh by 2050 and to 10,000 TWh by the year 2100 [261]. This projected increase in energy demand from AC systems would contribute significantly to greenhouse gases in the environment, which may hinder the achievement of the Paris Agreement target of <1.5 °C increase from pre-industrial period.

There is a need to transform the paradigm of AC and the future solution is likely to shift towards targeted body surface cooling, as opposed to cooling the space in the room [96,262,263]. If successful, targeted body surface cooling can maintain thermal comfort and Tb, with significantly less energy required and possibly, with no carbon emission. This paradigm shift in AC calls for the development of mobile, lightweight and energy-efficient body cooling systems by exploiting the heat exchange mechanisms of human thermoregulation [96,264,265]. This author developed a prototype PCD that relied on conductive, convective and evaporative heat exchange. The efficacy of this device was evaluated in 33 soldiers, who performed endurance marches in a climatic chamber, wearing bullet-proof vest and carrying about 35 kg of combat load. The participants marched on a treadmill at a speed of 4 km/h for 3 cycles of 45 min work and 15 min rest and the climatic chamber was programed to Tev 36 °C, 65% Rh and 800 W/m^2^ of simulated solar radiation. Using the body cooling device extended work tolerance by 1.2-fold to 2-fold and maintained peak Tc at <39.5 °C during the three work cycles [103]. The need for PCD is even greater with the threats posed by global warming and there are ongoing efforts to advance PCD technologies to meet current and future needs [210,257,263,264,266,267,268,269,270]. With technological advancements and the need to reduce carbon emission, it is conceivable that the concept of AC would shift from cooling the environment to cooling the body. Similar to the concept of heated blankets, the development of cooling blankets, scarfs, and vests, for example, can also help to maintain thermal comfort in static conditions, without the need to cool the entire room. Besides the innate thermoregulatory system and behavioral adaptations, heat stress management in the context of global warming would need to be reinforced with technological adaptations that promote effective body cooling using energy-efficient technologies that are also environmentally friendly.

### 3.7. Potential Adaptation Strategies in Public Health, Sport, and Occupational Settings

The impact of a significantly higher GMT would be the greatest in sub-populations who undertake physical work in outdoor environments. The burden of heat stress would be even greater if the work is conducted in protective suits that are made of materials that have limited or no permeability to sweat and air, and with the need to carry physical loads. For the general public, the innate drive for thermal comfort would encourage behavioral adjustments to reduce heat exposure and to promote heat dissipation, e.g., clothing, time and place of activity, and hydration. HA and raising public awareness on heat safety would need to be core components of public health strategies to protect the masses from environmental heat. One effective channel to promote public awareness on heat safey and a culture of HA from young age is through the school system. This channel has been used successfully in Junior and high schools in Australia for promoting behaviors to prevent skin cancer, e.g., “no hat, no play rule.” The same strategy can be applied to promote heat safety to the general public. Researchers also foresee a significant increase in the adoption of solar energy in residential and public infrastructures in the next 50 years.

In sport settings, a higher GMT would likely drive most indoor sport venues towards AC in the next few decades. However, AC is not a viable option for outdoor sports, which would need to rely on behavioral adaptations to overcome the effects of global warming. As already seen in the plans for the Tokyo 2020 Olympic Games (postponed to 2021), the timings and venues of most outdoor sports would need to be adjusted to be played in cooler places, seasons, and hours. Where applicable, body cooling technologies could be used to provide rapid body cooling to athletes during rest intervals (e.g., soccer, basketball, and rugby) and in events that involve multiple matches/bouts of competition within a short period of time (e.g., kayaking and sailing). It is possible that some outdoor sports would change to become indoor events, e.g., tennis, netball, short-put, long jump, and high jump). In the extreme, some rules in outdoor sports may need to be adjusted to protect athletes from the effects of environmental heat. For example, the introduction of compulsory breaks for body cooling at designated intervals may be necessary for endurance sports in the future.

The adaptation strategies to cope with global warming are more complex in outdoor occupational settings because of the wide variety of work tasks, environments, job routines, clothing, and equipment involved in different occupations. Examples of these occupations include agriculture, construction, outdoor laborers, military, law enforcement, and fire and emergency services. Besides HA and the basic heat-protective behaviors mentioned above, where practical, the working hours in outdoor occupations would need to be adjusted to operate in shorter shift cycles and in the cooler hours of the day. There should also be greater effort to replace human labor with machines and technologies to minimize the exposure of personnel to hot environment. For example, replacing foot patrol with street cameras in law enforcement and the use of robots to perform human tasks in farming, military, and emergency operations should be further exploited. Greater emphasis should be given to advancing the development of PCD that are light weight, portable, and energy efficient. Such a PCD would be most effective in maintaining Tb in outdoor occupations, especially when working in protective suits and clothing that are less or not permeable to sweat and air.

## 4. Conclusions

The impending threat of global warming will have significant impact on human life and would demand significant adjustments at the social, personal, industrial, and government levels to reduce carbon footprint in the next 10–20 years. The search for novel solutions to protect the environment from global warming needs to consider both the physical and physiological bases for human thermoregulation. The key concepts of human thermoregulation should form the bases for exploiting the potential of thermal plasticity to protect human life and functions in the context of global warming. For example, the benefits of hydration strategies, HA and physical fitness to enhance thermoregulation in the heat should be further exploited and implemented systematically to optimize the innate mechanisms of heat tolerance in education, sport, and occupation settings.

The innate capacity for heat dissipation, though limited, would serve as the foundation strategy to cope with elevated Tev. Other strategies involving behavioral adaptations and technological innovations can be designed around the human thermoregulatory system, to provide a holistic approach in protecting human life and functions from the impact of global warming. For example, the physical properties of heat transfer would be important considerations in the design of clothing materials to promote heat removal and the development of body cooling devices. Data on the metabolic demands of occupational tasks would also be valuable for guiding innovations on mechanization of physical labor. The combination of these human-centered innovations with behavioral adaptations would have great potential in delivering solutions to protect human survival and functions as the world faces an increasing GMT. This review serves to draw the attention of the global warming conversation to include the human and its physiology as part of the wider effort to protect the earth from the effects of global warming. There should be more integrated solutions between biologists and technologists, so that man and machine can enhance each other to bring about better solutions against the threat of global warming. Going back to the basics of appreciating the thermoregulatory system may serve as the foundation needed for future solution and human survival in a hotter environment.
